# Development of temperature controller-integrated portable HIFU driver for thermal coagulation

**DOI:** 10.1186/s12938-019-0697-3

**Published:** 2019-06-26

**Authors:** Suhyun Park, Ngot Thi Pham, Huu-Toan Huynh, Hyun Wook Kang

**Affiliations:** 1 School of Electrical and Electronics Engineering, Chung-Ang University, Seoul, 06974 South Korea; 2 Interdisciplinary Program of Marine-Bio, Electrical & Mechanical Engineering, Pukyong National University, Busan, 48513 South Korea; 3 Department of Electronic Engineering, Pukyong National University, Busan, 48513 South Korea; 4 Department of Biomedical Engineering and Center for Marine-Integrated Biotechnology (BK21 Plus), Pukyong National University, Busan, 48513 South Korea

**Keywords:** High-intensity focused ultrasound (HIFU), Temperature control, Proportional–integral–derivative (PID) controller, Coagulation

## Abstract

**Background:**

Temperature monitoring during high-intensity focused ultrasound (HIFU) therapy on tissue is essential to regulate the degree of thermal coagulation and to achieve the desired treatment outcomes eventually. The aim of the current study was to design and investigate the feasibility of a proportional–integral–derivative (PID) temperature controller-integrated portable HIFU driver for thermal coagulation.

**Methods:**

A portable HIFU driver was designed and operated at a maximum output voltage of 50 V with pulse-width modulation signals at 2 MHz. The temperature of ex vivo bovine liver tissue was monitored using a K-type thermocouple during the 2-MHz HIFU exposure.

**Results:**

The tissue temperature was maintained at 60 °C using a PID controller-integrated HIFU driver that modulated the output voltage during the 300-s HIFU exposure. The ex vivo testing demonstrated that the tissue temperature at the focal point approached the chosen temperature, i.e., 60 °C, within 70 s. The temperature was maintained with a deviation of less than 4 °C until the HIFU driver voltage was turned off at 300 s.

**Conclusions:**

The designed PID controller-integrated HIFU driver can be used as a small portable tool to regulate the tissue temperature in real time and achieve thermal coagulation via HIFU sonication.

## Background

Severe injury, i.e., trauma, is the third most common cause of mortality worldwide, and hemorrhage is responsible for 30 to 40% of mortality among trauma patients [1]. When physical injury occurs, immediate management of hemorrhage is thereby critical prior to the patients’ arrival to the hospital. A large number of hemostatic techniques have been clinically investigated to achieve rapid hemostasis and minimal complications, such as clamping, suturing, and argon plasma or laser coagulation [2, 3]. However, these methods are still invasive approaches; the location or geometry of the hemorrhage can limit the efficiency of such invasive methods. In addition, direct tissue contact often causes excessive heating and carbonized coagulum, possibly leading to collagenous fibrosis, i.e., scarring [4].

High-intensity focused ultrasound (HIFU) is a non-invasive therapeutic technology that utilizes acoustic energy (ultrasound waves) to induce thermal necrosis in the target tissue with minimal injury to the adjacent tissues [5, 6]. The clinical feasibility of HIFU has been evaluated in terms of thermal coagulation and ablation of tumors in the liver, prostate, breast, and thyroid [7–11]. Moreover, HIFU has been investigated to control acute hemorrhage in a non-invasive manner due to the rapid temperature increase and tissue coagulation [12–14]. Our previous study also demonstrated the feasibility of HIFU hemostasis on punctured blood vessels [2].

During HIFU treatment, the volumetric deposition of ultrasound energy within the tissue results in localized heating and leads to irreversible thermal damage. In soft tissue, thermal therapy utilizes a temperature of 60 °C to induce irreversible coagulation and to avoid necrosis of the tissue [6]. The clinical goal of HIFU therapy is to coagulate the target tissue area with no or minimal thermal injury to the surrounding healthy tissue. Thus, temperature regulation at the focal point during HIFU hemostasis is critical to control the degree of thermal coagulation at the bleeding sites. Previous studies focused on temperature monitoring during HIFU treatment with temperature sensors [15, 16]. A thermocouple (TC) is a temperature sensor that has a small diameter (0.2 mm), moderately low cost, high accuracy (~ 1 °C), and a relatively short response time, i.e., shorter than 1 s [16]. Thus, TCs could be useful tools to monitor and measure real-time tissue temperatures during HIFU therapy in spite of the invasiveness. Furthermore, a temperature feedback method can help maintain the tissue temperature constant during thermal treatment. The proportional–integral–derivative (PID) controller method is a well-known feedback strategy for thermometry [15, 17–19]. Previous studies have demonstrated the application of PID-integrated TCs for HIFU treatment in terms of real-time temperature control [15, 17, 18]. To facilitate point-of-care HIFU hemostasis, system portability is a critical factor. Although there have been several studies on portable HIFU drivers for hemostasis, none of them had real-time temperature control for thermal hemostasis [20–22].

In an attempt to develop a HIFU-assisted hemostasis approach, the current study focuses on the design of a portable HIFU driver integrated with a PID temperature controller and a TC for real-time temperature monitoring. The performance of the proposed HIFU driver was characterized in terms of waveform and acoustic pressure. The functionality of the integrated PID temperature controller was evaluated with ex vivo tissue during HIFU application. Both the extent of thermal coagulation and temperature feedback were validated by maintaining the tissue at the temperature required for irreversible coagulation.

## Materials and methods

### Design of HIFU system

**Fig. 1:**
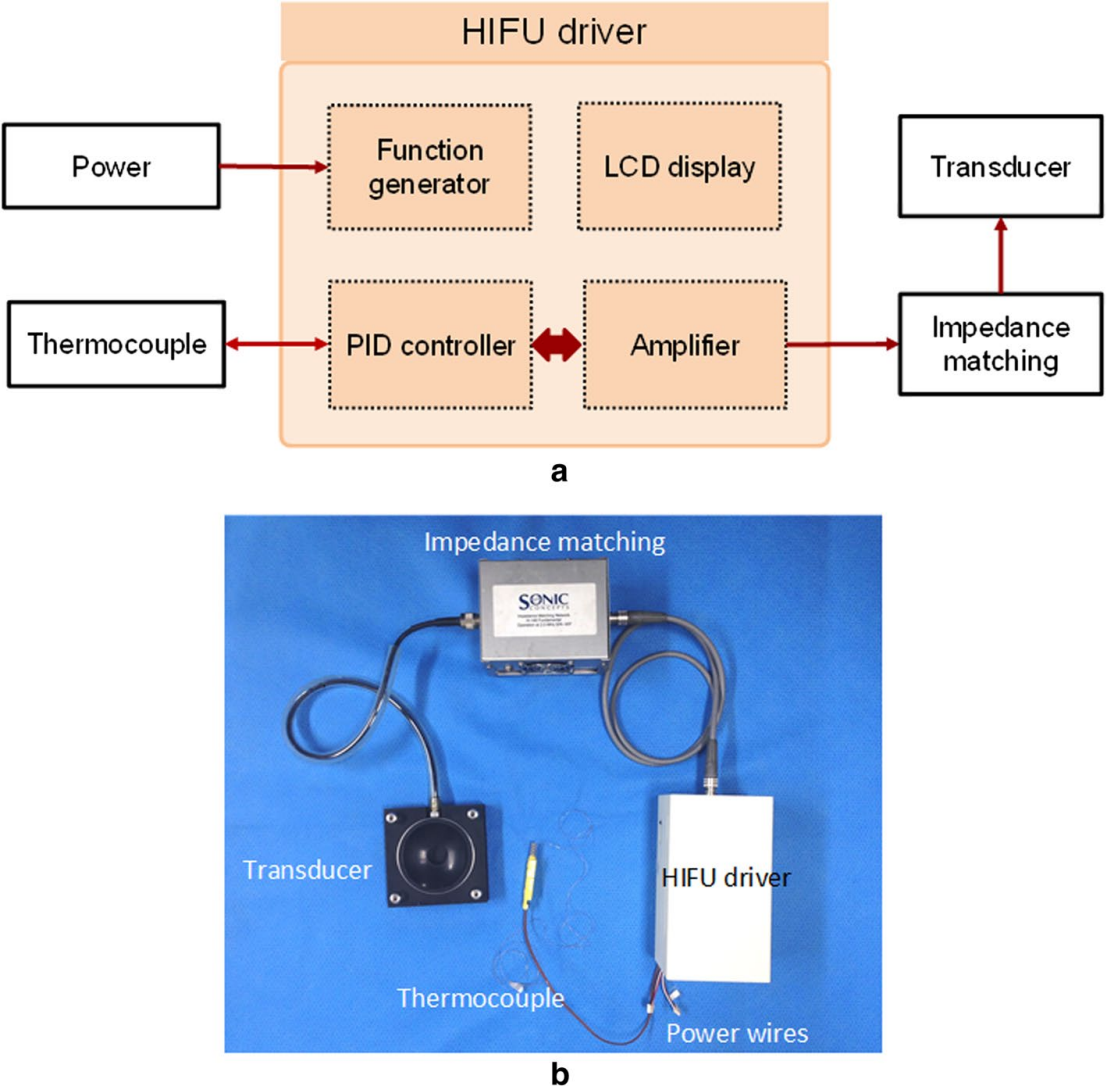
Proposed HIFU system: **a** schematic diagram and **b** photograph of system configuration

Figure 1a shows a schematic diagram of the proposed HIFU system. A HIFU driver consists of several functional blocks, including a function generator, an amplifier, a PID controller, and an LCD display. Figure 1b presents an actual image of the HIFU system. The HIFU driver was housed in a white plastic container (18 × 9 × 8 cm^3^). Each functional block of the HIFU driver was operated using an 8-bit microcontroller (ATmega328 p, Microchip Technology, Chandler, AZ). Arduino IDE was used to program and load the code into the microcontroller. A small LCD monitor (monochrome 128 × 64 OLED SSD1823, Adafruit Industry, New York City, NY) was installed to manually command several parameters of the HIFU driver (duty cycle, HIFU driver output voltage, working time, and desired temperature). The function generator utilized a CMOS synthesizer (AD9851, Analog Device, Norwood, MA) to produce pulse-width modulated (PWM) signals with duty cycles ranging from 20 to 80% at 2 MHz frequency. The high-voltage ultrasonic signal transmission, with a PWM voltage amplitude up to 50 V, was achieved using four high-speed dual MOSFET drivers (MD1213, Microchip Technology, Chandler, AZ) and N- and P-channel MOSFETs (TC6320, Microchip Technology, Chandler, AZ) [23–25]. A maximum amplitude of 50 V was selected in the present study to achieve the target temperature (around 60 °C) with minimum driver size. To control the temperature during HIFU coagulation, a PID controller was integrated into the HIFU driver. A K-type thermocouple (accuracy = ± 1.2 °C, TSL-101, Gaugeworld, Anyang, Korea) was connected to a thermocouple-to-digital converter (MAX31855, Maxim Integrated, San Jose, CA), which provided the measured temperature to the microcontroller.

For real-time temperature feedback, the PID controller was implemented with a public PID library that utilizes a relay tuning method [26–28]. The tissue temperature was maintained constant during modulation of the HIFU driver output. The PID controller calculated the error signal, which was the difference between the desired set point (SP) and process variables (PV). The error signal was minimized by changing the output signals. The control output, *u*(*t*), was corrected, based on the proportional, integral, and derivative terms as follows:

1 $$ u\left( t \right) = K_{\text{p}} e\left( t \right) + K_{\text{i}} \mathop \int \limits_{0}^{t} e\left( \tau \right){\text{d}}\tau + K_{\text{d}} \frac{{{\text{d}}e\left( t \right)}}{{{\text{d}}t}} $$

where *K*_p_, *K*_i_, and *K*_d_ are the proportional, integral, and derivative factors, respectively, *e*(*t*) = SP − PV(*t*) is the error, and t is the elapsed time (s). The initial values of *K*_p_, *K*_i_, and *K*_d_ factors were manually tuned by simply conducting iterative calculations to prevent the desired temperature from overshooting and to achieve fast rise time, high stability, and steady-state temperature with small errors during the exposure [28]. Thus, the HIFU driver voltage (*V*_out_) was continuously modulated in real time to maintain the desired temperature (i.e., 60 °C) in the tissue during HIFU exposure.

### Characterization of HIFU system

To demonstrate the feasibility of the HIFU driver operation, the HIFU driver was characterized in terms of output voltage and frequency spectrum. The output signals from the HIFU driver were acquired using an oscilloscope (Tektronix DPO3054, OR, USA) at various duty cycles (20, 50, and 80%) at a sampling rate of 2.5 G/s. The frequency spectrum was obtained using fast Fourier transform (FFT) and MATLAB (Math Works, Natick, MA, USA). The HIFU driver delivered the voltage to a HIFU transducer (H-148, Sonic Concept, Woodinville, UK) through an impedance matching network (50 Ω H-148, Sonic Concept, Woodinville, UK). The HIFU transducer had a center frequency of 2 MHz and focal length of 51.7 mm with an ellipsoidal focal beam (beam size of 0.36 × 5.72 mm at 400 W electrical power).

**Fig. 2:**
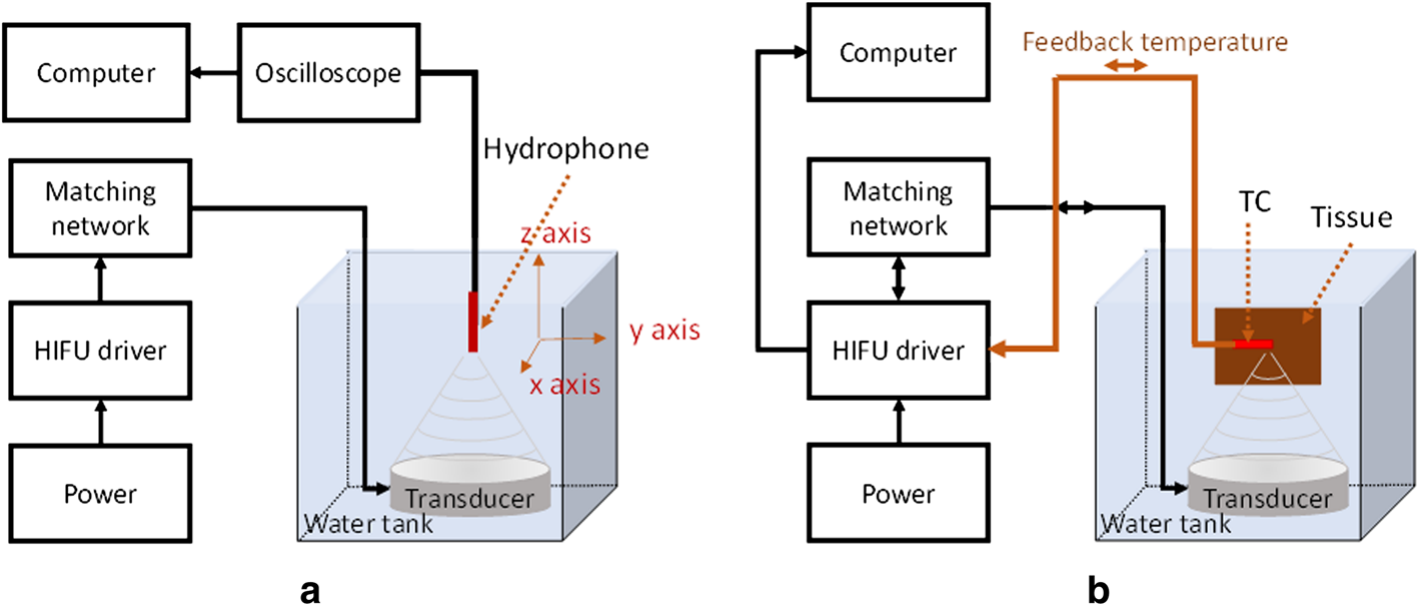
Experimental setup: **a** acoustic pressure measurement of a HIFU transducer and **b** ex vivo HIFU test with PID temperature controller

Figure 2a demonstrates the experimental setup for the acoustic characterization of a HIFU transducer. The acoustic pressure signals were obtained by scanning a needle hydrophone (0.2 mm needle, Precision Acoustics Ltd, Dorchester, UK) in a water tank. As the ultrasound source was operated at a 2 MHz PWM, the instantaneous acoustic pressure was calculated as follows:

2 $$ p\left( t \right) = \frac{V\left( t \right)}{{M\left( {f_{{2   {\text{MHz}}}} } \right)}} \;\;\left( {\text{MPa}} \right) $$

where *p*(*t*) is the acoustic pressure waveform (MPa), *V*(*t*) is the measured hydrophone voltage (mV), and *M*(*f*_2MHz_) is the hydrophone sensitivity at the acoustic frequency (sensitivity at 2 MHz = 64 mV/MPa). To ensure stable and safe operation of the hydrophone and to avoid high pressures (> 10 MPa) that could damage the hydrophone during the acoustic measurements, only low voltage levels from the HIFU driver (7–11 V) were tested. The transducer was placed in a water tank (inner area of 15 × 20 × 20 cm^3^) at 20 ± 0.5 °C (Fig. 2a). The hydrophone was attached to a three-axis computer controller positioning system and was moved along *x*-, *y*-, and *z*-axes to scan the acoustic signals, with a spatial resolution of 0.1 μm, at each output level to obtain the corresponding pressure values. The origin (*x* = *y* = *z* = 0) was taken at the focal point where the maximum acoustic signal occurred. To acquire the acoustic signals from the hydrophone, the oscilloscope had a sampling rate of 2.5 G/s.

### Experimental setup

Ex vivo bovine liver tissue was tested with the designed HIFU system to evaluate the temporal developments of temperature and degree of thermal damage during HIFU exposure. Figure 2b illustrates an experimental setup for the ex vivo HIFU treatment. The bovine liver tissue was acquired from a local slaughterhouse. Each specimen had dimensions 3 × 3 × 3 cm^3^ and was stored in saline solution at room temperature (20 °C) prior to testing. Each sample was firmly fixed in the water tank with the surface positioned at the beam focus. Square waveforms of frequency 2 MHz at 20, 50, and 80% duty cycles with 50 V output voltage (maximum voltage) were applied, and the HIFU transducer was turned on for 300 s. Then, a thermocouple was perpendicularly inserted into the tissue away from the focal point (~ 1 mm horizontally) and was connected to the HIFU driver to record real-time temperature variations using the PID temperature controller. For the PID controller, the maximum temperature at the focal point was set to 60 °C, the threshold for irreversible thermal damage in soft tissue. The initial tissue temperature was maintained at 20 ± 0.5 °C, i.e., no exposure. In this study, the initial values of the PID parameters were 5, 3, and 0.1 for *K*_p_, *K*_i_, and *K*_d_, respectively. The experiments were repeated five times for various ablation times (60, 120, 180, and 300 s). After HIFU testing, each sample was cross-sectioned in the longitudinal direction. Then, each cross section was analyzed using a digital camera. The coagulated regions (irreversible thermal denaturation) were defined as regions where discoloration, i.e., tan color, was observed in the tissue after HIFU exposure. The spatial extent of coagulation in the tested tissue was measured ten times using ImageJ (National Institute of the Health, Bethesda, MD) for quantitative evaluation. The statistical analysis, Mann–Whitney U test as a nonparametric method, represents insignificant *p* (< 0.05).

## Results

### Characteristics of HIFU driver

**Fig. 3:**
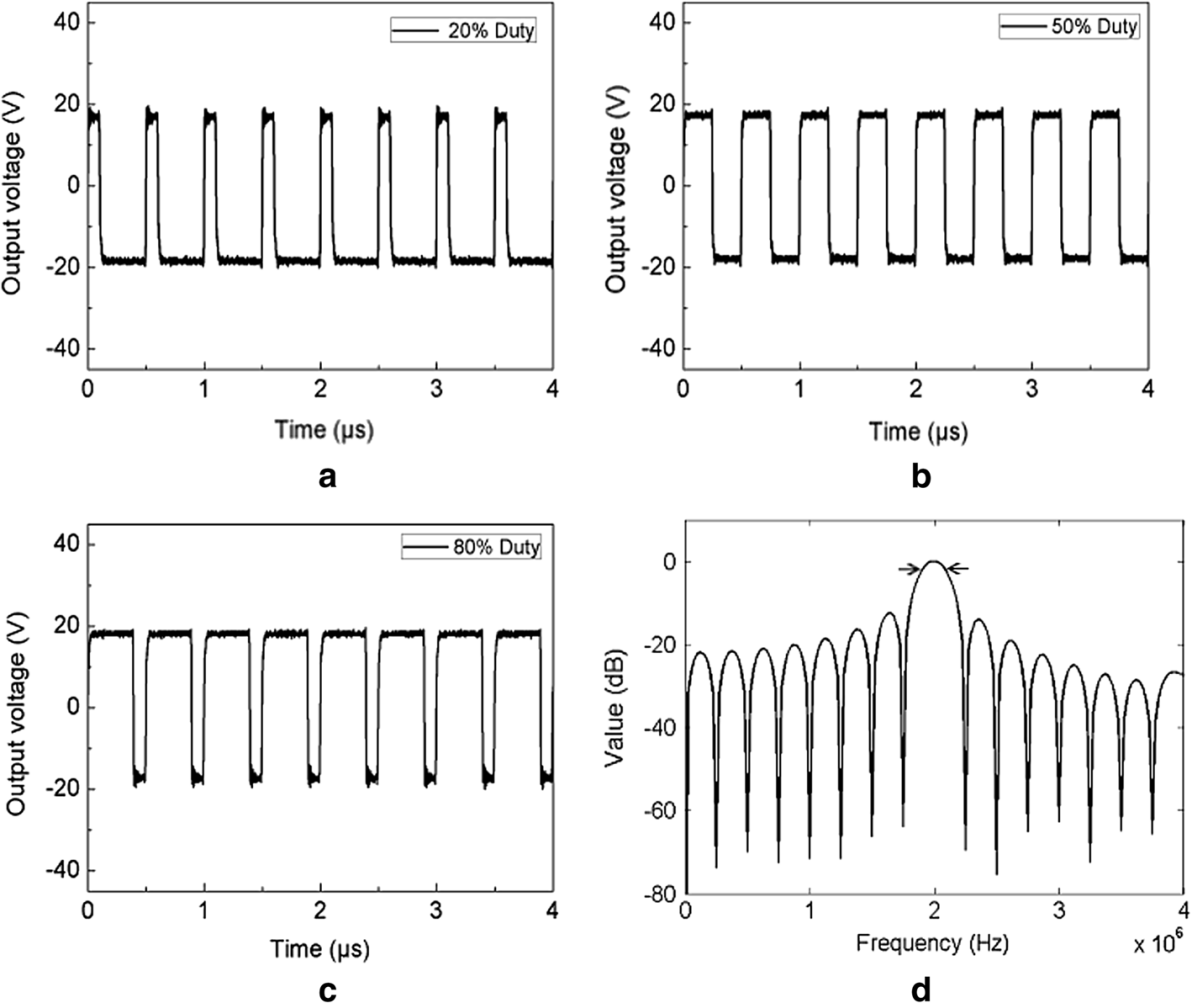
Output waveforms from HIFU driver measured at various duty cycles: **a** 20%, **b** 50%, and **c** 80%. **d** Normalized frequency spectrum of output waveform at 50% duty cycle (arrows represent − 3 dB bandwidth)

The designed HIFU driver was characterized in terms of output voltage and frequency spectrum (Fig. 3). The output waveforms of the HIFU driver were measured at 40 V output voltage and various duty cycles (20, 50, and 80%) on 4 µs. Regardless of the duty cycle, the overall output signals represented the square waveforms (symmetrical for positive and negative sides). The signals using a 50% duty cycle, as presented in Fig. 3b, yielded no oscillations at the edge of the waveform in the time domain and had a fast rise time (0.02 µs) between the push–pull cycles. On the other hand, the signals using 20% and 80% duty cycles, as shown in Fig. 3a, c, resulted in slight oscillations at the edges of the waveforms. The rise time for the 80% duty cycle was 13 and 6 times higher than those of the 20% and 50% duty cycles, respectively (i.e., 0.01, 0.02, and 0.13 µs for 20, 50, and 80%, respectively). Thus, the 50% duty cycle signals were chosen to deliver signals to the HIFU transducer. To acquire frequency spectrum, FFT was performed on 4-µs HIFU driver signals at 50% duty cycle. Figure 3d exhibits the frequency spectrum (dB scale) of the HIFU driver corresponding to the output waveforms in Fig. 3b. The highest normalized magnitude was found for the 2 MHz frequency with a − 3 dB bandwidth of 0.2 MHz, which was in good agreement with the center frequency of the HIFU driver. Thus, the HIFU driver could generate square wave signals at 2 MHz for 50% duty cycle.

### Characteristics of HIFU transducer

**Fig. 4:**
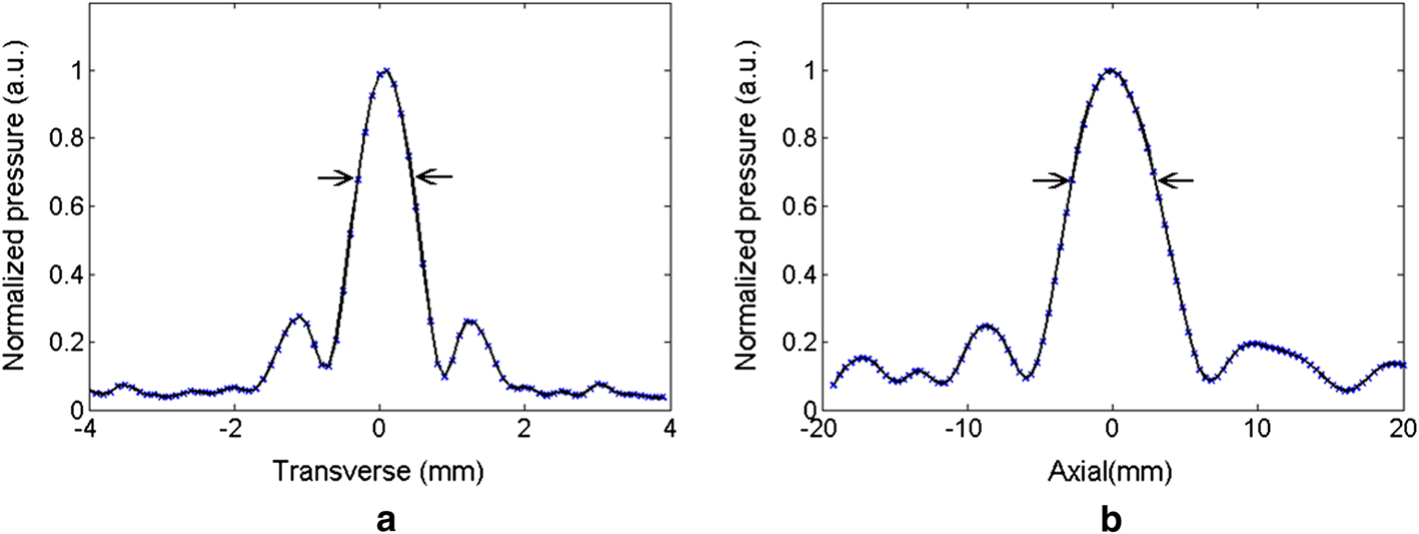
Spatial distribution of normalized acoustic pressure at the focal point of 2 MHz transducer in water: **a** transverse and **b** axial directions of beam propagation (arrows represent − 6 dB bandwidth)

**Fig. 5:**
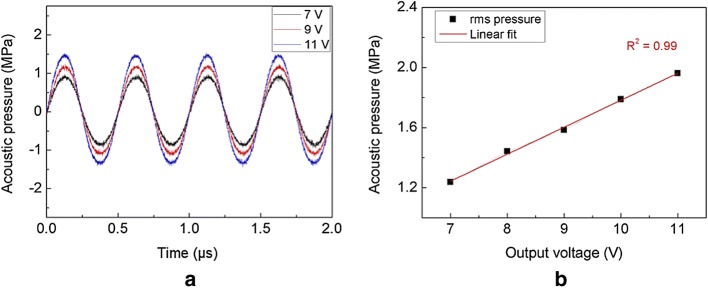
Characterization of acoustic pressure at focal point: **a** time-domain curve from various voltage levels of HIFU driver (7, 9 and 11 V) and **b** RMS pressure as a function of output voltage of 7–11 V

Figure 4 presents the spatial distribution of acoustic fields measured at the focal point of a HIFU transducer in water. The transversal beam profile is illustrated in Fig. 4a while the axial beam profile is shown in Fig. 4b across the focal plane of the HIFU transducer. Overall, the acoustic distribution followed an elliptical shape at the focal point, with a focused beam size of ~ 0.8 mm and ~ 6 mm in the transversal and axial directions, respectively, and at a − 6 dB beam dimension (full width at half maximum: FWHM). Regardless of the axial profile, the highest normalized acoustic pressure was found at the focal point and the pressure decreased significantly along the radial direction. Figure 5 demonstrates the acoustic pressure emitted from the transducer measured at the focal point under various voltage levels from the HIFU driver. Figure 5a exhibits the time-domain curve of the acoustic pressure at the focal point. Evidently, the acoustic pressure increased along with the output voltage levels. The corresponding RMS pressure was also estimated and showed a linear relationship with the output voltage from the HIFU driver (*R*^2^ = 0.99; Fig. 5b). According to Fig. 5b, a HIFU output voltage of 7–11 V resulted in RMS pressure levels of 1.34–1.95 MPa, respectively (conversion factor = 0.18 MPa/V).

### Ex vivo HIFU testing

**Fig. 6:**
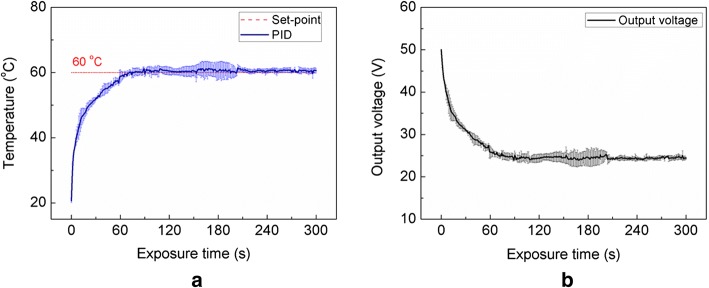
Temporal developments of **a** temperature and **b** output voltage from the HIFU driver at focal point during 300-s HIFU exposure with PID temperature controller. Predetermined temperature (set point) was set to 60 °C

A PID controller was employed to maintain a constant temperature during the entire HIFU treatment process in order to minimize unfavorable thermal injury to the peripheral tissue. Figure 6 shows the transient responses of temperature and output voltage during the 300-s HIFU exposure on ex vivo bovine liver tissue. The predetermined temperature was set to 60 °C, and the limit of the driving output voltage was selected as 50 V, based on previous findings. According to Fig. 6a, the PID-controlled temperature reached the target temperature within 70 s. Then, the target temperature was maintained at 60 °C with a deviation of less than 4 °C until the HIFU driver voltage was turned off at 300 s. Figure 6b presents the temporal changes of the output voltage from the HIFU driver during the HIFU exposure. The output voltage from the HIFU driver, i.e., the initial voltage of 50 V, was rapidly reduced in 70 s and attained a steady state at 25.1 ± 0.4 V, i.e., at an estimated RMS pressure of 4.51 MPa for the rest of the testing procedure, where the corresponding tissue temperature was 60 ± 4 °C.

**Fig. 7:**
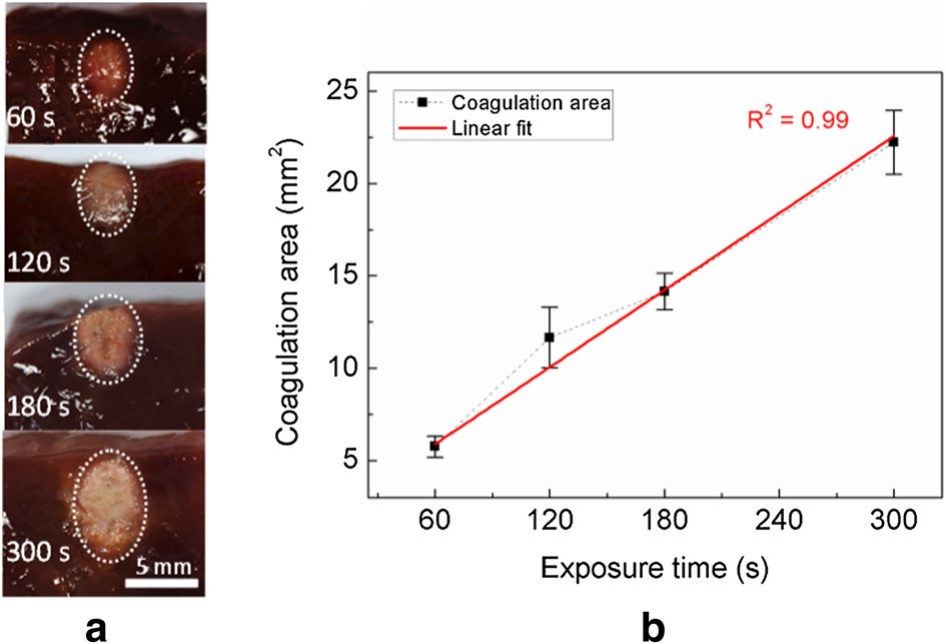
Thermal coagulation in ex vivo bovine liver tissue during 300-s HIFU exposure with PID controller: **a** transverse cross-sectional images of coagulated tissue captured at various exposure times (60, 120, 180 and 300 s) and **b** coagulation area as a function of exposure time (*N* = 5, red line represents a linear fitting of coagulation area (*R*^2^ = 0.99))

Figure 7 exhibits the development of irreversible thermal denaturation during various HIFU treatment times (60, 120, 180, and 300 s) with a PID controller and a 50-V HIFU driver. Figure 7a shows a series of transverse cross-sectional images of the coagulated tissue captured at various exposure times. Owing to the acoustic beam distribution, as shown in Fig. 4, the cross-sectional images showed ellipsoidal coagulative responses. Apparently, the degree of thermal coagulation increased with exposure time. The tissue coagulation was quantified as a function of exposure time in Fig. 7b. The coagulation area increased linearly with exposure time; the red line represents a linear fitting (*R*^2^ = 0.99). The tissue coagulation area was 22.2 ± 1.7 mm^2^ for a 300 s exposure time, which was 74, 48, and 36% larger than those at 60 s (5.8 ± 0.6 mm^2^), 120 s (11.7 ± 1.6 mm^2^), and 180 s (14.2 ± 1.0 mm^2^), respectively. It is noted that the consistent maintenance of the target temperature at 60 °C resulted in a predictable expansion of thermal coagulation in the tissue during the PID controller-assisted HIFU exposure.

## Discussion

The goal of this study was to demonstrate the feasibility of a PID controller-integrated portable HIFU driver for real-time temperature regulation during HIFU application. The overall control of the HIFU driver, including ultrasound waveform generator and PID controller, was implemented using a microcontroller programmed in Arduino code. As controls of the functional blocks in the HIFU driver were easily accessible and programmable, the current results show that the proposed approach is suitable for further development of the portable HIFU system for thermal hemostasis. The HIFU driver was operated at a maximum output voltage of 50 V, which was delivered to a HIFU transducer. Based upon the linear correlation from Fig. 5b, the corresponding RMS pressure at 50 V could be assumed to be 8.99 MPa [29]. The PID controller was integrated with the HIFU driver in a portable device of dimensions 18 × 9 × 8 cm^3^, which was evaluated with ex vivo tissue to assess the temperature feedback function for clinical applications. The PID-assisted HIFU application thereby induced a temperature of 60 °C at the focal point in the tissue; this temperature was maintained by modulating the HIFU voltage during the exposure, as shown in Fig. 6. The degree of tissue coagulation increased linearly with exposure time without carbonization, implicating the predictability of thermal coagulation during the PID-integrated HIFU treatment.

Acoustic characterization is significant for precise prediction of thermal effects in tissue as well as quantitative evaluation of the efficacy and safety of the HIFU treatment [5]. The current study quantified the acoustic pressure by characterizing the focal beam of the HIFU transducer. However, all the pressure levels were measured at low voltage levels (7–11 V) to avoid any physical damage to the hydrophone due to the generation of strong acoustic pressures. Instead of direct measurements, a linear conversion factor of 0.18 MPa/V was used to estimate the pressure levels at high voltage levels, according to previous studies [30]. However, further experiments are required to confirm the linear relationship between the RMS pressure and high voltages (up to 50 V) using a fiber optic probe hydrophone or a radiation force balance method [31, 32].

Defining the extent of coagulation can be a critical factor in thermal hemostasis, depending on both the temperature of the tissue and exposure time to acoustic energy [5, 33]. In this study, the real-time target temperature was maintained at 60 °C by regulating the HIFU driver voltage output during the treatment (Fig. 6). Figure 7 shows the results of the tissue coagulation in ex vivo liver tissue when the HIFU exposure time was increased to linearly augment the volumetric heat deposition in the tissue. The area of tissue coagulation at 300-s exposure (22.2 ± 1.7 mm^2^) was 3.8 times larger than that at 60 s (5.8 ± 0.6 mm^2^). PID-integrated HIFU driver was able to apply and maintain the desired tissue temperature. In addition, as the ultrasound beam shape in the hydrophone measurement (Fig. 4) matched the shape of the coagulated tissue in Fig. 7a, it was confirmed that the focal field of the HIFU system was controllable and able to have sharp transitions between the treated and untreated areas. Although the current study tested a single preset temperature to demonstrate the feasibility of regulating both the tissue temperature and irreversible coagulation, the entire range of controllable temperatures during HIFU therapy still needs to be investigated to explore maximum therapeutic capability.

The HIFU instantaneous intensity (*I* in W/cm^2^) can be estimated using the following equation [34]:

3 $$ I = p^{2} /\rho \cdot c $$

where *p* is the acoustic pressure (Pa), *ρ* is the water density (1000 kg/s m^3^ at 20 °C), and *c* is the speed of sound in water (1481 m/s at 20 °C). The maximum acoustic intensity could be 5460 W/cm^2^ in case the maximum possible acoustic pressure was assumed to be 8.99 MPa. This can vary with HIFU transducers, but it is possible that the current HIFU system delivered an acoustic intensity of up to 5460 W/cm^2^ from the designed 50 V-HIFU driver, which is still below the maximum intensity of the transducer, i.e., 46,161 W/cm^2^ from H-148, according to the manufacturer’s specifications.

The implemented PID controller continuously adjusts the HIFU voltage levels to maintain the desired temperature at the focal point in tissue during exposure (Fig. 6). The quality of the PID controller typically depends on proper regulation of various parameters. To prevent any overshooting or undershooting of the temperature as well as to minimize steady-state errors, the main parameters of the PID controller, i.e., K_p_, K_i_, and K_d_, should be optimized iteratively. This optimization resulted in a steady-state error of less than 4 °C (Fig. 6a). The coagulated tissue using the PID parameters hardly showed any carbonization (Fig. 7a), implicating that the PID temperature controller was properly programmed and functioned correctly. However, as various soft tissues have different thermal responses [35], further testing of the PID factors with various tissue types is necessary to determine appropriate values of *K*_p_, *K*_i_, and *K*_d_ and to establish a versatile platform for non-invasive thermal hemostasis.

Despite the feasibility of a portable PID controller-integrated HIFU driver to regulate tissue temperature and thermal coagulation during treatment, a number of limitations remain for practical applications. Although the eventual goal of our study was HIFU application for thermal hemostasis, the current feasibility study mainly focused on validating the performance of the designed PID controller-integrated HIFU driver with ex vivo tissue. In an effort to prove the concept of HIFU hemostasis, the proposed method should be assessed and confirmed with blood vessels and blood as shown in the previous study [2]. For the sake of experimental reliability, the current study positioned the tip of the TC near the focal area of the HIFU transducer to monitor real-time tissue temperature. However, this temperature monitoring technique is still minimally invasive, which may limit its clinical applications. In addition, the measured temperature might depend on the position of the thermocouple, primarily on the account of effects from viscous heating and acoustic oscillations [36, 37]. These effects have a less significant impact on the thermocouple measurements when the tissue temperature is assessed farther away from the axis of the focal point [36, 38]. Hence, to ensure real-time temperature monitoring of the proposed treatment, in the current experiments, the thermocouple was positioned ~ 1 mm horizontally away from the axial axis of the focal point. However, the viscous effect needs to be further investigated to verify any additive thermal effects during tissue temperature monitoring, for example, using an infrared camera or a thin-film thermocouple [36, 37].

The current system and its acoustic pressure range (1.34–8.99 MPa) may be applicable for treatment of superficial hemorrhages or benign tumors shallowly situated under the skin such as thyroid nodules and breast fibroadenomas. However, the system has a limitation of invasive single-point measurement of temperature. Previous studies have shown that temperature monitoring during HIFU treatment is possible with non-invasive spatial temperature monitoring tools, such as ultrasound and magnetic resonance imaging (MRI) [6, 39, 40]. Ultrasound imaging can track temperature changes by analyzing various temperature-dependent properties such as speed of sound, attenuation, and echo strain of tissue [19, 41]. MRI thermometry can measure the interstitial tissue temperature by utilizing temperature-sensitive MR parameters [6, 40]. Recent non-invasive temperature monitoring techniques, including 3D IR thermal imaging and ultrasound thermometries using passive mapping and shear wave elastography [42–46], can also be incorporated with the proposed HIFU system. However, the portability of the HIFU system should be a priority for the emergent use of the thermal hemostasis. Further design of the HIFU driver with an array transducer will be studied to obtain acoustic powers high enough to treat deeply located hemorrhages. Following the design and performance improvements, in vivo animal testing in rodent and leporine models will be conducted to elucidate acute and chronic tissue responses to HIFU sonication in terms of degree of thermal injury and hemostasis. The eventual aim is to assess the efficacy and safety of the PID-integrated HIFU system for clinical translation.

## Conclusions

The current study demonstrated the feasibility of a PID temperature controller-integrated HIFU driver for thermal coagulation. The designed driver resulted in a HIFU system that was portable as well as able to regulate tissue temperature in real time for predictable tissue coagulation. The PID controller-integrated HIFU driver is a feasible tool to control both tissue temperature and thermal coagulation precisely during HIFU treatment. Further improvements on non-invasive temperature monitoring and acoustic power generation will be pursued to broaden the therapeutic applications of the proposed PID feedback HIFU system.

## Data Availability

The datasets generated and/or analyzed during the current study are available from the corresponding author on reasonable request.
